# Dental age estimation in forensic odontology: a systematic review comparing traditional methods and artificial intelligence approaches

**DOI:** 10.3389/fdmed.2026.1882454

**Published:** 2026-08-26

**Authors:** Firdaous Lakhaouaja, Ana García Navarro

**Affiliations:** Faculty of Health Sciences, Department of Dentistry, Universidad Europea de Valencia, Valencia, Spain

**Keywords:** AI forensic dentistry, artificial intelligence, dental age estimation, forensic odontology, machine learning

## Abstract

Dental age estimation is a fundamental tool in forensic odontology, with important applications in human identification and legal contexts. This systematic review evaluated the accuracy, objectivity, and reproducibility of traditional dental age estimation methods compared with artificial intelligence–based approaches. A search was conducted in PubMed, Scopus, and Web of Science including studies published between 2015 and 2025, selecting 16 studies that analysed traditional methods, machine learning, and deep learning models. Artificial intelligence–based approaches, particularly machine learning and deep learning, demonstrated higher accuracy with lower mean absolute error (MAE) values compared with traditional methods such as Demirjian, Cameriere, and Kvaal. In addition, these models reduced intra- and inter-observer variability, improving objectivity and reproducibility. Despite these promising results, methodological heterogeneity and the need for large, well-structured datasets remain important limitations. Artificial intelligence represents a valuable and promising tool for improving dental age estimation in forensic practice.

## 1 Introduction

Dental age estimation represents a fundamental tool in forensic odontology, with important applications in human identification and legal contexts (1). Traditionally, age estimation has relied on radiographic methods such as those proposed by Demirjian, Cameriere, and Kvaal, which are widely used in both clinical and medico-legal practice (2–4). However, these methods present several limitations, including observer subjectivity, inter- and intra-examiner variability, and the influence of population-specific factors, all of which may affect the accuracy and reproducibility of the results (2–8).

In recent years, artificial intelligence has emerged as a promising alternative, enabling automated analysis of radiographic images through machine learning and deep learning techniques (9–12). Convolutional neural networks have demonstrated a high capacity to analyze medical images and identify complex patterns in an automated manner (13–15). These approaches have the potential to improve accuracy, reduce the mean absolute error, and enhance the objectivity of age estimation (16, 17).

Nevertheless, the available evidence is characterized by considerable methodological heterogeneity, which limits the comparability of results and their applicability in forensic practice (18–20).

The aim of this systematic review was to compare the accuracy, objectivity, and reproducibility of traditional dental age estimation methods with those based on artificial intelligence.

The specific objectives of this systematic review were:

1. To analyze studies published between 2015 and 2025 on artificial intelligence–based dental age estimation for forensic purposes.
2. To identify the advantages and limitations of traditional dental age estimation methods.
3. To describe the main artificial intelligence algorithms used in dental age estimation.
4. To evaluate the strengths and limitations of artificial intelligence–based approaches.
5. To propose recommendations for the practical application of artificial intelligence in forensic dental age estimation.

## 2 Materials and methods

This systematic review was conducted in accordance with the Preferred Reporting Items for Systematic Reviews and Meta-Analyses (PRISMA) guidelines (22), with the aim of identifying and analyzing studies assessing dental age estimation using both traditional methods and artificial intelligence–based approaches in the field of forensic odontology. This systematic review was not prospectively registered in PROSPERO or any other systematic review registry. This absence of protocol registration is acknowledged as a limitation and should be considered when interpreting the findings.

### 2.1 Focus question (PICO)

The research question was formulated according to the structured PICO framework:

**Population (P):** Human individuals, either living or deceased, whose age was estimated through dental analysis based on radiographic imaging, including panoramic radiographs and cone-beam computed tomography (CBCT).
**Intervention (I):** Artificial intelligence-based approaches for dental age estimation, including machine learning, deep learning, and convolutional neural networks (CNN).
**Comparison (C):** Conventional dental age estimation methods, primarily Demirjian, Cameriere, and Kvaal.
**Outcomes (O):**
  • O1: diagnostic accuracy, assessed by the mean absolute error (MAE).
  • O2: correlation between estimated dental age and chronological age.
  • O3: objectivity and inter-observer variability.
  • O4: reproducibility of the results.

### 2.2 Eligibility criteria

The eligibility criteria were defined as follows:

#### 2.2.1 Inclusion criteria

Studies were included if they met the following variables were extracted:

- Published between 2015 and 2025.
- Conducted on human subjects.
- Based on dental radiographic imaging, including panoramic radiographs or cone-beam computed tomography (CBCT).
- Applied artificial intelligence–based methods.
- Reported quantitative measures of accuracy, primarily mean absolute error (MAE).
- Studies evaluating traditional dental age estimation methods when they allowed direct comparison with artificial intelligence-based approaches.
- Published in English, Spanish, or French.

#### 2.2.2 Exclusion criteria

Studies were excluded if they:

- Were not related to dental age estimation (e.g., skeletal maturity or archaeological studies).
- Included pathological conditions affecting dental development.
- Were reviews, meta-analyses, case reports, or *in vitro* studies.

### 2.3 Sources of information and search strategy

A systematic search was conducted in three major electronic databases (PubMed, Scopus, and Web of Science) using keywords related to forensic odontology, dental age estimation, traditional methods, and artificial intelligence. The search strategy included terms such as: “dental age estimation”, “age determination by teeth”, “forensic dental age”, “forensic odontology”, “artificial intelligence”, “machine learning”, “deep learning”, “neural network*”, “convolutional neural networks”, “CNN”, “Demirjian”, “Cameriere”, “Kvaal”, “traditional method*”, “accuracy”, “reproducibility”, “objectivity”, “correlation”, and “validity”. These terms were combined using Boolean operators (AND, OR, NOT) and controlled vocabulary terms (MeSH Terms in PubMed; Title/Abstract and TS fields in Web of Science) to ensure broad and accurate identification of relevant studies. The PubMed search strategy was conducted using the following query:((“Dental Age Estimation”[Title/Abstract] OR “Age Determination by Teeth”[MeSH] OR “forensic dental age”[Title/Abstract] OR “forensic odontology”[Title/Abstract]) AND (“Artificial Intelligence”[MeSH] OR “machine learning”[Title/Abstract] OR “deep learning”[Title/Abstract] OR “neural network*”[Title/Abstract] OR CNN[Title/Abstract]) AND (Demirjian[Title/Abstract] OR Cameriere[Title/Abstract] OR Kvaal[Title/Abstract] OR “traditional method*”[Title/Abstract]) AND (accuracy[Title/Abstract] OR reproducibility[Title/Abstract] OR objectivity[Title/Abstract] OR correlation[Title/Abstract] OR validity[Title/Abstract])). In Web of Science, the search strategy was performed using the Topic field (TS), as follows: (((((((([TS = (“forensic odontology”)] AND TS = (“age determination”)) OR TS = (“age estimation”)) AND TS = (“artificial intelligence”)) OR TS = (“deep learning”)) OR TS = (“convolutional neural networks”)) OR TS = (cnn)) AND TS = (“demirjian method”)) OR TS = (“kvaal method”)) OR TS = (“cameriere method”)). To identify any potentially eligible studies that might have been missed during the initial search, the reference lists of all included articles were manually reviewed. Duplicate studies were removed before screening, and additional manual searches were carried out when necessary to ensure comprehensive coverage of the available literature.

### 2.4 Study selection process

Study selection was performed independently by two reviewers in three sequential phases:

1. Title screening, to exclude clearly irrelevant studies;
2. Abstract screening, to assess eligibility according to the predefined criteria;
3. Full-text review, to confirm final inclusion.

Disagreements were resolved by consensus. Duplicate records were removed prior to screening.

### 2.5 Data extraction

Data extraction was independently performed by two reviewers using a standardized data collection form. Extracted variables included study design, population characteristics, imaging modality, sample size, AI algorithm, outcome measures, reference method and main findings. Any disagreements were resolved through discussion and consensus.

The sfollowing variables were extracted:

Primary outcome:
  • Accuracy of dental age estimation, assessed by the mean absolute error (MAE) and its standard deviation.
Secondary outcomes:
  • Type of artificial intelligence algorithm used.
  • Reference traditional method (Demirjian, Cameriere, or Kvaal).
  • Type of radiographic imaging (panoramic radiography or CBCT).

The extracted data were organized into comparative tables.

### 2.6 Quality and risk of bias assessment

Two reviewers (FL, AGN) independently evaluated the methodological quality of the included studies. The methodological quality and risk of bias of the included studies were independently evaluated by two reviewers using the Newcastle-Ottawa Scale (21).

The Newcastle-Ottawa Scale was selected because all included studies were observational in nature. Although AI-specific assessment frameworks such as PROBAST, CLAIM and QUADAS-AI have been proposed, none has been specifically developed or universally adopted for systematic reviews focusing on artificial intelligence applications in forensic dentistry. The Newcastle-Ottawa Scale remains one of the most widely used instruments for assessing methodological quality in observational studies.

Studies scoring more than 6 stars were considered to have a low risk of bias, whereas those scoring 6 or fewer stars were classified as high risk. Any disagreements were resolved by consensus.

### 2.7 Data synthesis

A qualitative and quantitative synthesis of the results was performed.

The primary outcome measure was the mean absolute error (MAE). When possible, weighted means were calculated based on sample size.

Studies were grouped into three categories:

- traditional methods;
- machine learning models;
- deep learning models.

Due to the methodological heterogeneity among the included studies, a meta-analysis was not feasible. Therefore, the results are presented as a structured narrative synthesis.

## 3 Results

### 3.1 Study selection

**Figure 1:**
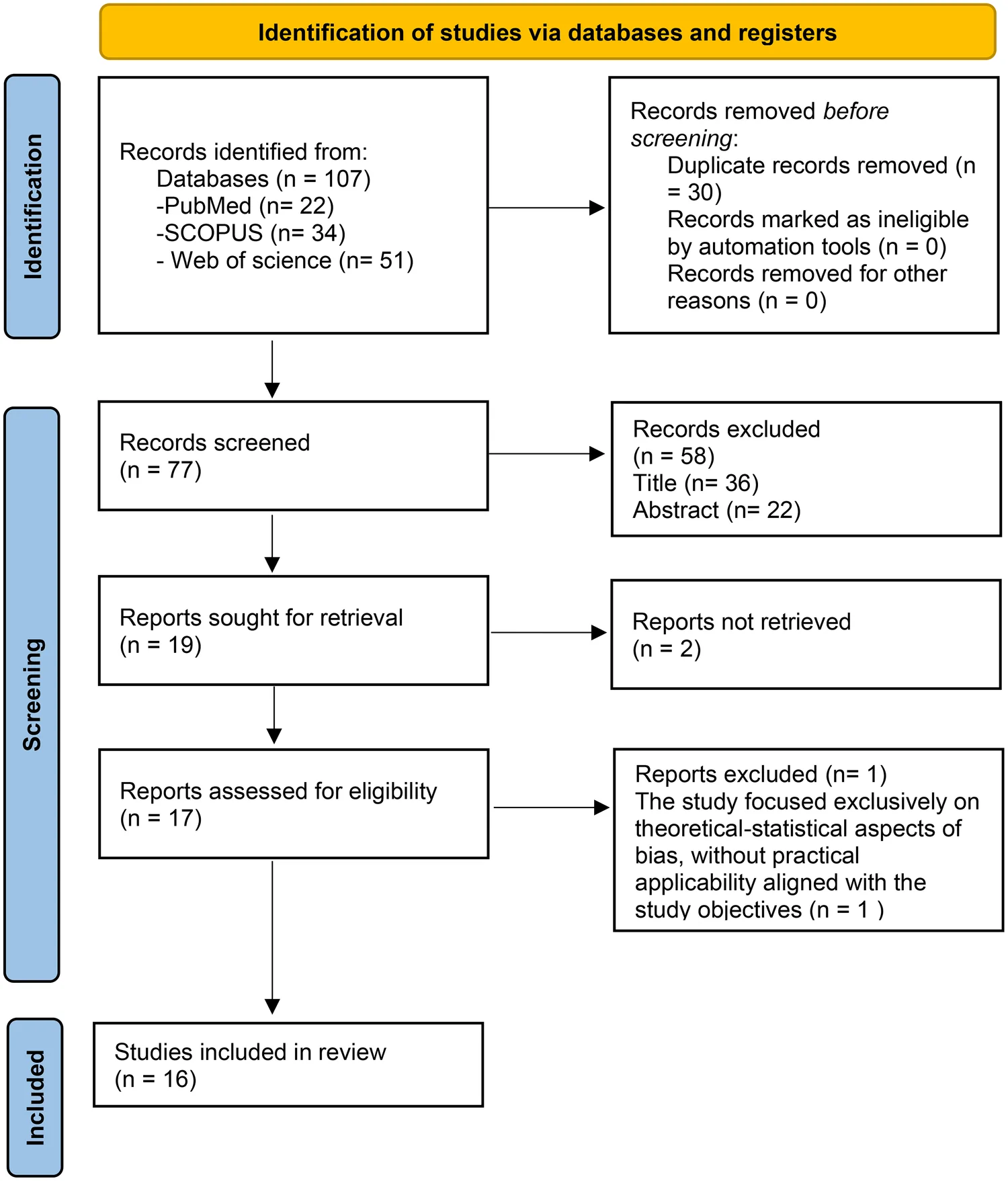
PRISMA 2020 flow diagram illustrating the study selection process. Adapted from Page et al. (2021) (22), PRISMA 2020 Statement.

A total of 107 records were identified through electronic database searching. After removal of 30 duplicate records, 77 records remained for screening. During title and abstract screening, 58 records were excluded, 19 reports were sought for retrieval, of which 2 could not be obtained in full text. Seventeen full-text articles were assessed for eligibility, and one was excluded. Therefore, 16 studies were ultimately included in the systematic review (Figure 1).

### 3.2 Analysis of the characteristics of the reviewed studies

The 16 included studies evaluated dental age estimation using radiographic imaging, primarily panoramic radiographs and, to a lesser extent, cone-beam computed tomography (CBCT) (23–28). Studies were classified into three methodological groups: traditional methods (*n* = 4), machine learning models (*n* = 6), and deep learning models (*n* = 6) (23–25, 27–31). Studies employing hybrid approaches that combined machine learning and deep learning techniques were categorized according to the predominant methodological component. Classification was based on the primary analytical approach described by the authors and the methodology predominantly used to generate the reported outcomes.

All studies had a retrospective design, based on dental imaging datasets obtained in clinical settings (23–25). The study populations consisted mainly of children and adolescents, although some studies also included young adults (26–28). Sample sizes varied considerably, ranging from fewer than 500 to more than 3,000 individuals.

The most used traditional methods were Demirjian (23, 30), Cameriere (24, 32), and Kvaal (28, 29). Regarding artificial intelligence approaches, the most frequently applied algorithms included Random Forest, Support Vector Machine, K-Nearest Neighbors, and convolutional neural networks (CNN) (23–26, 33–37). Panoramic radiography was the predominant imaging modality among the included studies (24–27, 34–38), whereas CBCT was used less frequently and primarily in studies evaluating traditional approaches (27, 28). The main characteristics of the included studies, including methodological approach, imaging modality, study population, and outcome measures, are summarized in Table 1.

**Table 1:** Characteristics of the included studies.

Variable	Traditional methods	Machine learning	Deep learning	Total
Number of studies	4	6	6	16
Imaging modality				
Panoramic radiography (OPG)	2	6	6	14*
CBCT	2	1	0	3*
Study design				
Retrospective	4	6	6	16
Type of method applied				
Demirjian	2	3	2	7
Cameriere	2	2	0	4
Kvaal	2	1	0	3
Molar development (2nd/3rd molars)	0	1	3	4
AI model type				
Random forest	0	3	0	3
Support Vector Machine (SVM)	0	2	0	2
Decision tree	0	1	0	1
K-Nearest Neighbors (KNN)	0	1	0	1
Ensemble/hybrid models	0	3	0	3
Convolutional Neural Networks (CNN)	0	0	4	4
VGG16	0	0	1	1
Advanced architectures (Transformer/pipelines)	0	0	3	3
Study population				
Children	2	3	2	7
Children and adolescents	2	3	3	8
Adolescents/young adults	0	0	1	1
Sample size				
<500 subjects	2	2	2	6
500–1,000 subjects	1	2	2	5
>1,000 subjects	1	2	2	5
Dental variables analyzed				
Developmental stages (Demirjian)	2	3	2	7
Open apices (Cameriere)	2	2	0	4
Pulp-tooth ratio (Kvaal)	2	1	0	3
Molar development	0	1	3	4
Outcome measures				
MAE	1	5	4	10
RMSE	0	3	3	6
R^2^ (correlation)	1	3	1	5
Accuracy	0	2	3	5
Sensitivity/specificity	0	1	2	3

OPG, orthopantomography; CBCT, cone-beam computed tomography; MAE, mean absolute error; RMSE, root mean square error; CNN, convolutional neural network; SVM, support vector machine; KNN, k-nearest neighbors.

* One study used both OPG and CBCT, therefore, the total number of imaging modality observations exceeds the total number of included studies.

### 3.3 Risk of bias

All included studies were retrospective observational studies and were evaluated using the Newcastle-Ottawa Scale (21). Overall methodological quality was considered moderate, with scores ranging from 4 to 7 stars (23–38). The main limitations identified were limited population representativeness, absence of formal sample size calculation, and insufficient control of confounding factors (23–38). The risk of bias assessment of the included studies is presented in Table 2.

**Table 2:** Risk of bias assessment of included observational studies using the Newcastle–Ottawa scale (adapted for cross-sectional studies) (21).

	Representativeness of sample	Sample size	Non-respondents	Exposure ascertainment	Control of confounders	Outcome assessment	Statistical analysis	Reporting of precision	Total score
Różyło-Kalinowska et al., (27)	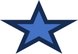	—	—	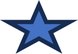	—	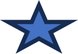	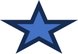	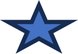	5
Çelik et al., (28)	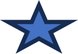	—	—	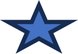	—	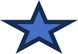	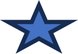	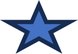	5
Pereira de Sousa et al., (29)	—	—	—	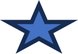	—	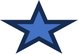	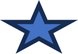	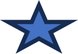	4
Abuabara et al., (30)	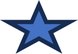	—	—	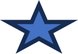	—	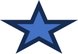	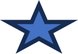	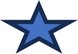	5
Abuabara et al., (31)	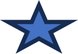	—	—	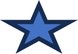	—	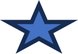	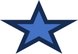	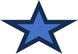	5
Galibourg et al., (23)	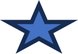	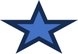	—	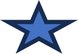	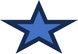	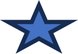	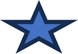	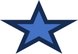	7
Shen et al., (24)	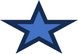	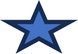	—	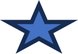	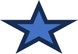	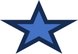	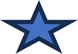	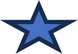	7
Shen et al., (25)	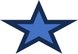	—	—	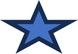	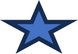	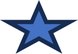	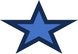	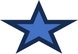	6
Zeng et al., (26)	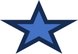	—	—	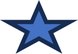	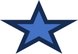	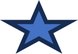	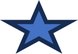	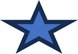	6
Simsek et al., (38)	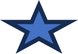	—	—	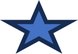	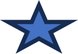	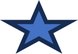	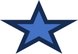	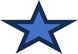	6
El-Desouky et al., (32)	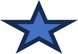	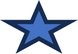	—	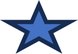	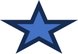	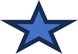	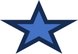	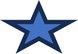	7
Nushi et al., (34)	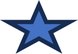	—	—	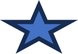	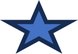	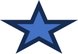	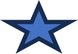	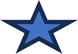	6
Upalananda et al., (35)	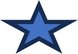	—	—	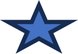	—	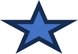	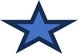	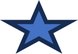	5
Pintana et al., (36)	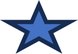	—	—	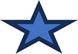	—	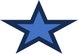	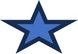	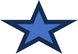	5
Li et al., (37)	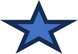	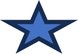	—	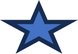	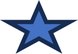	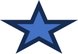	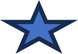	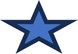	7
Shi et al., (33)	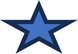	—	—	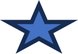	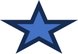	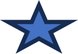	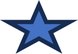	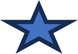	6

### 3.4 Synthesis of results

#### 3.4.1 Accuracy of dental age estimation methods

Traditional methods showed variable accuracy depending on the method used and the population analyzed. The Cameriere method applied to CBCT demonstrated MAE values ranging from 0.73 to 0.77 years (27); however, its performance was influenced by population-specific factors, with deviations observed in certain groups (32).

The Kvaal method showed lower accuracy, with MAE values of up to 5.68 years and low correlation (R^2^ = 0.335) (29), findings that were also confirmed in CBCT-based studies (28). In contrast, the Demirjian method demonstrated intermediate accuracy (MAE ≈ 1.34 years), which improved significantly when combined with artificial intelligence models, reducing the error to approximately 0.75 years (30). Machine learning models demonstrated a substantial improvement in accuracy, Galibourg et al. (23) reported MAE values below 0.811 years compared to 1.107 years for the traditional method, while Shen et al. (24, 25) obtained values between 0.47 and 0.49-years using algorithms such as KNN and SVM. Similarly, models based on molar development analysis achieved MAE values ranging from 0.42 to 0.72 years (26). Deep learning models yielded the best overall performance. Simsek et al. (38) reported errors below 6 months, whereas Shi et al. (33) achieved an MAE of approximately 0.72 years using fully automated systems. Other studies reported MAE values between 1.15 and 1.28 years (37).

In contrast, generative artificial intelligence models showed lower accuracy, with MAE values ranging from 1.57 to 2.05 years (31).

The lower performance observed with large language models should be interpreted cautiously. Unlike convolutional neural networks and image-based machine learning systems, large language models are not specifically designed for radiographic image analysis and therefore should not be considered directly comparable to image-based AI approaches.

**Figure 2:**
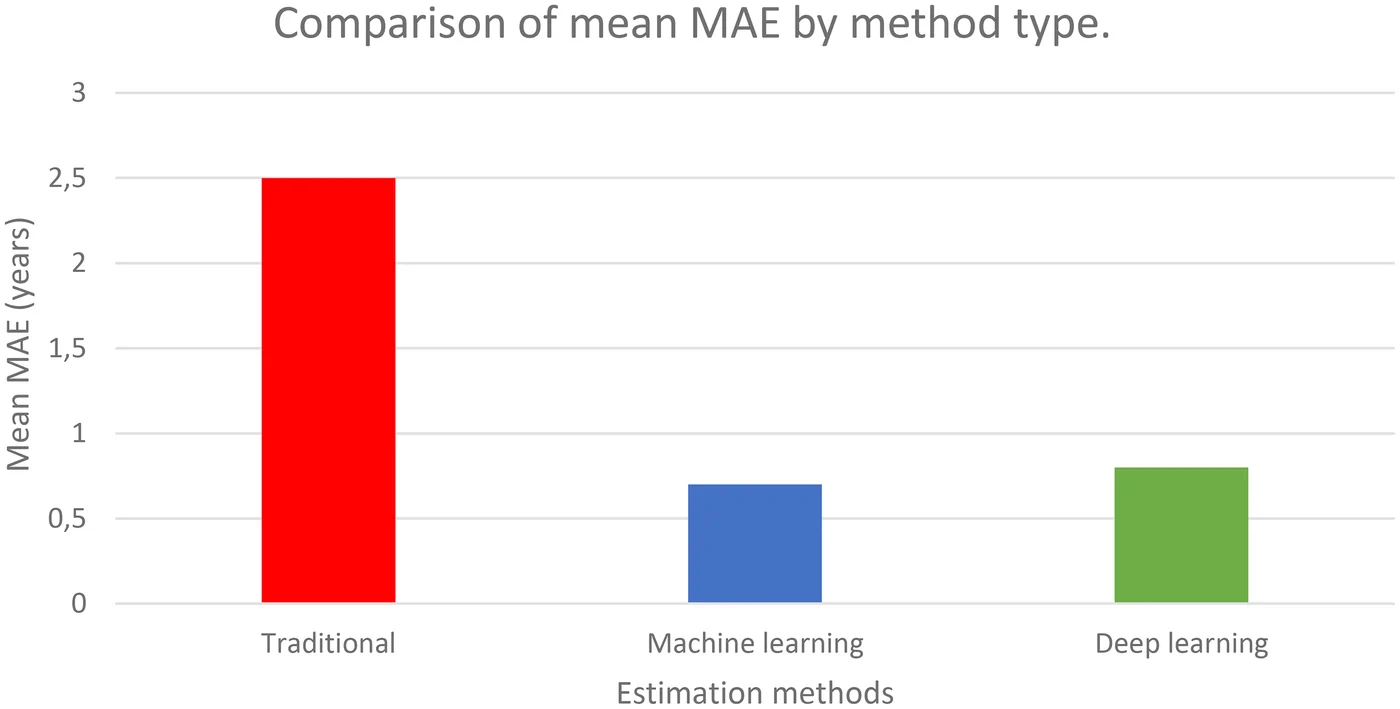
Comparison of mean absolute error (MAE) values among traditional methods, machine learning, and deep learning approaches. Lower MAE values are generally associated with greater age estimation accuracy. Results should be interpreted cautiously due to methodological heterogeneity among included studies.

As shown in Figure 2, artificial intelligence–based methods demonstrated lower mean absolute error (MAE) values compared to traditional methods, indicating higher accuracy in dental age estimation (23–38). The values presented in Figure 2 represent descriptive averages derived from the included studies and should not be interpreted as pooled estimates. A detailed comparison of accuracy is presented in Table 3.

**Table 3:** Characteristics and performance of the included studies.

Author (Year)	Evaluated method	Approach type	Primary metric (key outcome)	Main result	Scientific contribution
Różyło-Kalinowska et al., (27)	Cameriere (CBCT)	Traditional	MAE: 0.73–0.77 years	High accuracy in pediatric population	Validation using CBCT
Çelik et al., (28)	Kvaal (CBCT)	Traditional	Not reported	Moderate accuracy	Limited performance in adults
Pereira de Sousa et al., (29)	Kvaal vs ML (XGBoost, RF, SVR)	Traditional vs ML	MAE: 5.68 vs 4.65 years	ML improves Kvaal method	Integration of radiomics and ML
Abuabara et al., (30)	Demirjian vs ML (RF, GB, KNN)	Traditional vs ML	MAE: 1.34 vs 0.75 years	∼44% error reduction	Clinical validation of ML
Abuabara et al., (31)	LLM (ChatGPT, Gemini, DeepSeek)	Generative AI	MAE: 1.57–2.05 years	Low performance	Limitations of generative AI
Galibourg et al., (23)	Demirjian vs ML (10 algorithms)	Traditional vs ML	MAE: 1.107 vs <0.811 years	ML outperforms traditional method	Large multicenter study
Shen et al., (24	Demirjian/Cameriere + ML (DT, BRR, KNN)	ML comparative	MAE: 0.982–0.473 years	Best model: KNN + Cameriere	Influence of algorithm selection
Shen et al., (25)	Cameriere vs ML (SVM, RF, LR)	Traditional vs ML	MAE: 0.846 vs 0.489 years	ML improves Cameriere method	Impact of preprocessing
Zeng et al., (26)	M2–M3 + ML (RF, Voting, SVM)	Machine learning	MAE: 0.42–0.72 years	High accuracy across age groups	Use of molar development
Simsek et al., (38)	DL + ML (Swin, ExtraTrees)	Hybrid deep learning	MAE: 5–7 months	Very high accuracy	Advanced hybrid pipeline
El-Desouky et al., (32)	Cameriere (original vs adapted)	Traditional	Error: −0.59 → ±0.1 years	Improved after adaptation	Population-specific calibration
Nushi et al., (34)	CNN (VGG16) vs classical methods	Deep learning	Accuracy > 90%	High classification accuracy	Stage classification
Upalananda et al., (35	Demirjian vs CNN	Traditional vs DL	Sensitivity: 0.45 vs 0.65–0.74	DL improves sensitivity	Diagnostic balance
Pintana et al., (36)	Detector + CNN (ResNet50)	Deep learning	Accuracy: 99.5%	Fully automated system	Detection + classification
Li et al., (37)	CNN (multiple models)	Deep learning	MAE: 1.15–1.28 years	Stable performance	Sex-based validation
Shi et al., (33)	YOLOv3 + SOS-Net + regression	Automated deep learning	MAE: ∼0.72 years	Fully integrated pipeline	End-to-end automation

MAE, mean absolute error; ML, machine learning; DL, deep learning; CNN, convolutional neural network; RF, random forest; GB, gradient boosting; KNN, k-nearest neighbors; SVM, support vector machine; SVR, support vector regression; DT, decision tree; BRR, Bayesian ridge regression; LR, linear regression; CBCT, cone-beam computed tomography; LLM, large language model.

#### 3.4.2 Correlation between estimated and chronological age

Traditional methods showed a moderate correlation with chronological age. The Cameriere method demonstrated minor deviations (27, 32), whereas the Kvaal method showed low correlation (R^2^ = 0.335) (29). In contrast, artificial intelligence–based models demonstrated stronger correlations. Machine learning approaches achieved R^2^ values of up to 0.94 (24, 25) while hybrid deep learning models reported errors below 0.5 years, indicating a high level of agreement with chronological age (38).

#### 3.4.3 Objectivity and inter-observer variability

Traditional methods showed a high degree of operator dependence, particularly in the Demirjian method (23, 30). The Cameriere method demonstrated higher reproducibility (27), whereas the Kvaal method showed greater variability due to the need for multiple manual measurements (28, 29).

Artificial intelligence approaches significantly reduced this variability. Machine learning models decreased subjectivity by using quantitative variables (24–26) while deep learning models virtually eliminated human intervention (33). In this context, convolutional neural network–based models achieved accuracies above 90%, and fully automated systems reached values of up to 99.5% (36), effectively minimizing inter-observer variability.

#### 3.4.4 Reproducibility and automation

Traditional methods showed limited automation and lower reproducibility (27–29).

In contrast, machine learning models improved the consistency of results (23–26), while deep learning models enabled full automation of the process (33, 37). Automated systems integrated detection, classification, and age estimation into a single workflow, significantly enhancing both reproducibility and analytical efficiency (36, 37).

#### 3.4.5 Overall summary of results

Overall, artificial intelligence–based methods generally demonstrated lower MAE values and improved reproducibility compared with traditional approaches (23–38). Machine learning models improved traditional methods by reducing estimation error, whereas deep learning approaches enabled full automation of the diagnostic process (33–37).

However, these models still present limitations related to data availability, lack of methodological standardization, and challenges in generalizing results across different populations (33, 34).

## 4 Discussion

The findings of this systematic review suggest that artificial intelligence–based methods were generally associated with lower MAE values than traditional approaches, as well as decreased inter- and intra-observer variability.

Among the included studies, machine learning models, such as those described by Galibourg et al. (23) and Shen et al. (24, 25) consistently achieved MAE values below 1 year, outperforming traditional methods.

Similarly, Abuabara et al. (30) reported a significant reduction in estimation error when machine learning algorithms were applied to the Demirjian method. In the case of deep learning models, studies by Simsek et al. (38) and Shi et al. (33) reported even lower errors, in some cases below 0.5 years. Notably, the study evaluating large language models reported considerably lower performance than that observed for image-based machine learning and deep learning systems, reinforcing the importance of distinguishing between all the AI different applications.

These findings are consistent with recent literature. Sivri et al. (39) demonstrated higher accuracy of convolutional neural network models compared to the Demirjian method, while Han et al. (40) highlighted that machine learning approaches can match or even surpass traditional methods when trained on large and well-structured datasets. In addition, recent reviews have emphasized that artificial intelligence can reduce estimation error and improve consistency (8). The superior performance of artificial intelligence–based models may be explained by their ability to analyze complete radiographic images and integrate multiple variables simultaneously. Unlike traditional approaches, which rely on predefined criteria, deep learning models can identify complex patterns that are not easily detectable through visual assessment (33, 34, 36).

Nevertheless, the apparently superior performance of AI-based approaches should not be interpreted as final evidence of clinical or forensic superiority. Many of the included studies relied on internally validated datasets and lacked independent external validation, which will result in optimistic performance estimations when compared with real-world forensic applications.

Furthermore, automation significantly reduces operator dependency, thereby minimizing inter- and intra-examiner variability (23–26, 33). This aspect is particularly relevant in forensic contexts, where objectivity and reproducibility are essential for the validity of expert assessments.

Traditional methods such as Demirjian, Cameriere, and Kvaal remain widely used due to their simplicity, low cost, and ease of application. However, the studies included in this review highlight important limitations, particularly operator dependency and population variability, which may negatively affect accuracy and reproducibility (23, 27, 29).

### 4.1 Population generalizability

Despite the promising performance of artificial intelligence, these results should be interpreted with caution. Model performance is highly dependent on the quality, size, and diversity of the training datasets, as observed in the included studies (23–26, 33–37).

A major limitation identified across the included research concerns population generalizability. Most of the AI models were developed and validated using geographically restricted datasets, including Brazilian, Chinese, Turkish, Egyptian and Spanish populations. Consequently, the model performance may decrease when it is applied to individuals from different ethnic or demographic backgrounds. This issue is very relevant in forensic contexts, where the geographical and ancestry origins of unidentified individuals are unknown.

These observations are in line with recent literature, which suggests that while artificial intelligence can enhance dental age estimation, its effectiveness depends on data quality and methodological design (8, 39, 40).

Considerable heterogeneity was observed across the included studies. Sources of heterogeneity included population age range, imaging modality, ethnicity, anatomical structures analyzed, AI architecture, reference standards, validation strategies and outcome metrics. These differences limited directly the comparison between studies and prevented quantitative meta-analyses.

In addition, the heterogeneity among the included studies in terms of imaging techniques, algorithms, and sample characteristics prevented the performance of a meta-analysis; therefore, the findings are presented descriptively.

### 4.2 Forensic implications

Predictive accuracy by itself is insufficient to justify practical implementation. Forensic expert opinions require reproducibility, transparency, legal defensibility and validation under real world conditions. Therefore, despite the promising performance of AI-based systems, addicional evidence is required before these methods could be considered a substitute for established age estimation procedures.

### 4.3 Ethical and medicolegal considerations

The integration of AI into forensic age estimation also raises ethical and medicolegal concerns. Potential algorithmic bias, lack of standardized regulatory frameworks, absence of explainability and the possibility of erroneous decisions in legal contexts remain important challenges. Future research should address these issues in order to ensure responsible implementation of AI-assisted forensic tools.

From a clinical and forensic perspective, the integration of artificial intelligence may improve the reliability and efficiency of dental age estimation, particularly in contexts where precision is critical, such as legal procedures or mass disaster victim identification.

### 4.4 Limitations of the review

This review has several limitations that should be considered when interpreting the findings: First, the search strategy was restricted to three databases (Scopus, PubMed and Web Of Science) and to studies published in French, English or Spanish which may have resulted in the exclusion of relevant evidence published in other languages. Second, grey literature, dissertations, conference proceedings and technical reports were not included. And finally, publication bias may have contributed to an overrepresentation of studies reporting positive results for AI-based approaches.

An additional limitation concerns the use of the Newcastle-Ottawa Scale, which was originally developed for observational studies and may not fully capture AI-specific methodological issues such as external validation, algorithm transparency, model overfitting and reporting quality.

### 4.5 Future directions

Future research should prioritize the development of large multicenter datasets, standardized reporting frameworks, external validation procedures and explainable artificial intelligence models. These measures are essential to improve reliability, transparency and acceptance of AI-assisted age estimation in forensic practice. Furthermore, prospective studies will involve diverse populations in order to establish the true clinical and forensic applicability of these technologies.

However, these technologies should be considered as a support tool rather than a replacement for clinical judgment. Further validation through prospective studies and diverse populations is required before widespread implementation.

## 5 Conclusion

Overall, artificial intelligence–based methods generally demonstrated lower MAE values and improved the reproducibility compared with traditional approaches. However, direct comparisons should be interpreted cautiously because of substantial methodological heterogeneity across studies. Therefore, although AI-based approaches show considerable promise, further external validation, methodological standardization and multicenter studies are required before widespread implementation in forensic practice.

## Data Availability

The original contributions presented in the study are included in the article/Supplementary Material, further inquiries can be directed to the corresponding author/s.
